# Isorhapontigenin (ISO) inhibited cell transformation by inducing G0/G1 phase arrest via increasing MKP-1 mRNA stability

**DOI:** 10.18632/oncotarget.1872

**Published:** 2014-03-26

**Authors:** Guangxun Gao, Liang Chen, Jingxia Li, Dongyun Zhang, Yong Fang, Haishan Huang, Xiequn Chen, Chuanshu Huang

**Affiliations:** ^1^ Nelson Institute of Environmental Medicine, New York University School of Medicine,Tuxedo, NY, USA; ^2^ Department of Hematology, Xijing Hospital, Fourth Military Medical University,Xi'an, Shaanxi, China

**Keywords:** Isorhapontigenin, MKP-1, Transformation, Chemoprevention, Cyclin D1

## Abstract

The cancer chemopreventive property of Chinese herb new isolate isorhapontigenin (ISO) and mechanisms underlying its activity have never been explored. Here we demonstrated that ISO treatment with various concentrations for 3 weeks could dramatically inhibit TPA/EGF-induced cell transformation of Cl41 cells in Soft Agar assay, whereas co-incubation of cells with ISO at the same concentrations could elicit G0/G1 cell-cycle arrest without redundant cytotoxic effects on non-transformed cells. Further studies showed that ISO treatment resulted in cyclin D1 downregulation in dose- and time-dependent manner. Our results indicated that ISO regulated cyclin D1 at transcription level via targeting JNK/C-Jun/AP-1 activation. Moreover, we found that ISO-inhibited JNK/C-Jun/AP-1 activation was mediated by both upregulation of MKP-1 expression through increasing its mRNA stability and deactivating MKK7. Most importantly, MKP-1 knockdown could attenuate ISO-mediated suppression of JNK/C-Jun activation and cyclin D1 expression, as well as G0/G1 cell cycle arrest and cell transformation inhibition, while ectopic expression of FLAG-cyclin D1 T286A mutant also reversed ISO-induced G0/G1 cell-cycle arrest and inhibition of cell transformation. Our results demonstrated that ISO is a promising chemopreventive agent via upregulating *mkp-1* mRNA stability, which is distinct from its cancer therapeutic effect with downregulation of XIAP and cyclin D1 expression.

## INTRODUCTION

Albeit intensive efforts that have focused on therapeutics development, cancer is still a leading health problem worldwide [1]. In past few decades, the concept of chemoprevention has emerged and become a new strategy in fighting against cancers [2-4]. World Health Organization (WHO) indicates that at least 30% of all cancer deaths are preventable [5]. Since diverse phytochemicals were reported to interfere with a specific stage of the carcinogenic process, numerous efforts have been devoted to identifying phytochemicals and phytochemical-derived agents with cancer preventive properties [4, 6-7].

Isorhapontigenin (ISO) is a new derivative of stilbene isolated from the Chinese herb *Gnetum Cleistostachyum* [8]. ISO was also recently identified from wine grapes that are the main dietary source of stilbene [9]. Despite several investigations on biological properties of ISO such as its antioxidant effect [10-11], the anti-cancer activity of this compound has not been evaluated until quite recently, and it has been found that ISO triggers apoptosis in multiple human cancer cell lines [12-13]. Mechanistically, ISO treatment is shown to downregulate XIAP and cyclin D1 expression by promoting transcription factor Sp1 protein degradation [12-13]. However, ISO chemopreventive effects have not been explored thus far. In the current study, therefore, we using TPA/EGF-induced mouse Cl41 cell transformation model sought to investigate the potential chemopreventive activity of ISO and molecular mechanisms underlying its activity. We found that ISO was capable of inhibiting TPA/EGF-induced cell transformation with induction of G0/G1 cell-cycle arrest by downregulating cyclin D1 transcription via both upregulating MKP-1 expression and deactivating MKK7/JNK cascade.

## RESULTS

### ISO inhibited cell transformation and induced G0/G1 cell-cycle arrest with no redundant cytotoxic effects on non-transformed cells

**Figure 1:**
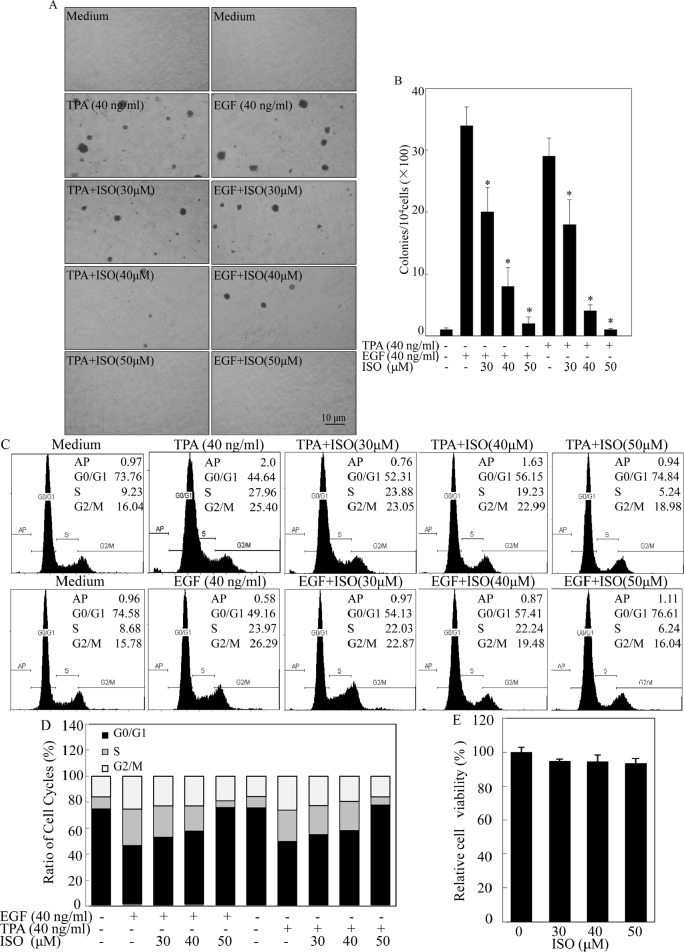
ISO inhibited cell transformation and induced G0/G1 cell-cycle arrest with no redundant cytotoxic effects on non-transformed Cl41 cells (A) Representative images of colonies of Cl41 cells in soft agar assay. Cells were co-treated with TPA/EGF (40 ng /ml) and various concentrations of ISO as indicated. (B) The number of colonies was counted under microscopy in soft agar after 3 weeks and the results were presented as colonies per 10,000 cells from three independent experiments. The asterisk (*) indicates a significant difference in Cl41 cells treated with different doses of ISO compared with cell treated with TPA or EGF alone respectively (*P*<0.05). (C and D) Flow cytometric analysis of cell cycle distribution. Cl41 cells were pretreated with various concentrations of ISO for 30 min and then co-incubated with ISO and TPA/EGF (40 ng /ml) as described in “Materials and Methods”. Data was represent one of three different experiments. (E) Cl41 cells were treated with various concentrations of ISO (30, 40 or 50 μM) for 48 hours. Percentage of cell viability was expressed as relative to medium control in triplicate.

To investigate the potential chemopreventive activity of ISO, TPA/EGF-induced Cl41 cell transformation model was employed. Given that ISO could reduce cell viability in T24T bladder cancer cells with an approximate IC50 of 55 μM [12], we thus treated mouse epidermal Cl41 cells with ISO in concentrations of 30, 40, and 50 μM with exposure to TPA/EGF. As shown in Figs. 1A and 1B, co-incubation of cells with ISO for 3 weeks significantly inhibited TPA/EGF-induced anchorage-independent colony formation in a dose-dependent manner in Cl41 cells, indicating that ISO is a potential preventive agent. To further explore whether the inhibitory effect of ISO on cell transformation is due to its induction of apoptosis and/or cell cycle arrest, high-resolution flow cytometry analysis of PI-stained nuclei was performed. The data revealed that treatment of cells with ISO at the same concentrations for 48 hours was capable of significantly reversing TPA/EGF-induced G1/S phase progression in a dose-dependent manner, whereas almost no apoptosis was triggered under the same experimental condition (Figs. 1C and 1D). Considering that an ideal chemopreventive agent should be able to impart apoptotic/anti proliferative effects specifically in carcinogen/tumor promoter-treated cells without affecting normal cells [6], we thus evaluate the cytotoxic effect of ISO on normal non-transformed Cl41 cells using ATPase assay. The data showed that ISO did not exert any notable growth inhibition at the concentration range 30-50 μM at 48 hours after the treatment (Fig. 1E). These results demonstrated that ISO could remarkably inhibit the growth of transformed Cl41 cells via arresting G1/S progression without redundant cytotoxic effects on non-transformed cells.

### ISO downregulation of cyclin D1 expression was responsible for its induction of G0/G1 phase arrest and inhibition of cell transformation

**Figure 2:**
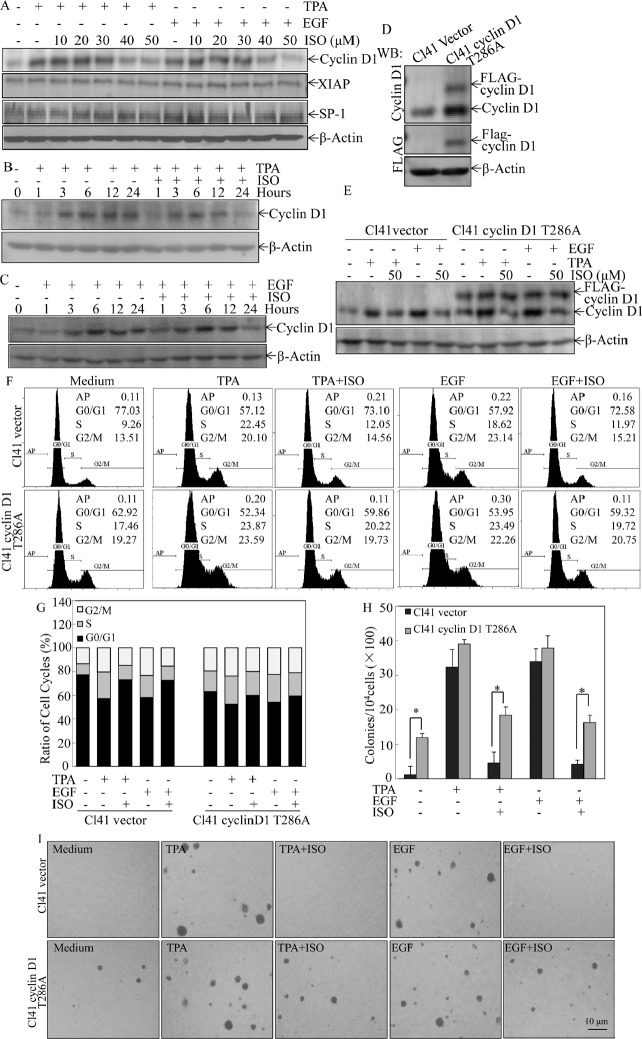
Downregulation of cyclin D1 expression mediated the inhibition of colony formation and induction of G0/G1 phase arrest following ISO treatment (A-C) Cl41 cells were pretreated with indicated doses of ISO for 30 min and then co-treated with ISO and TPA/EGF (40 ng /ml) for 24 hours (A) or pretreated with 50 μM ISO for 30 min and then co-incubated with ISO and TPA/EGF for the indicated time periods (B and C). The protein expression levels were determined by Western blot, and β-actin levels were used to as protein loading control. Data was representative one of three independent experiments. (D and E) The FLAG-cyclin D1 expression levels were determined by Western blot in Cl41 cells transfected with plasmid encoding FLAG-tagged cyclin D1 T286A mutant and its vector control. Antibodies specifically against cyclin D1 and FLAG were used, respectively, to detect the protein as indicated in the figure (D). Transfectants were treated with TPA/EGF (40 ng /ml) alone, or pretreated with 50 μM ISO for 30 min and then co-incubated with ISO and TPA/EGF for 24 hours. Antibody specifically against cyclin D1 was used to determine cyclin D1 expression (E). (F and G) Cl41 cells were treated with TPA/EGF (40 ng /ml) alone, or pretreated with 50 μM ISO for 30 min and then co-incubated with ISO and TPA/EGF for 24 hours. Cell cycle distribution was determined by flow cytometric analysis. Data represent one of three different experiments. (H and I) Cells stably transfected with vector control or FLAG-cyclinD1 T286A were treated with medium containing either DMSO or TPA/EGF (40 ng /ml) with or without exposure to ISO (50 μM) for 3 weeks. Representative images of colonies of transfectants in soft agar assay were presented (I). Colonies were counted after 3 weeks and the results were presented as colonies per 10,000 cells from three independent experiments (H). The asterisk (*) indicates a significant difference of colony number between Cl41 FLAG-cyclinD1 T286A cells and Cl41 vector cells (*P*<0.05).

To delineate the molecular basis of ISO-induced inhibition of cell transformation and reversion of G1/S phase transition due to TPA or EGF treatment, we performed Western blot analysis to probe the molecular targets that could be potentially regulated by this phytochemical. Cells were pretreated with ISO in different concentrations for 30 min and then co-incubated with TPA/EGF for 24 hours. The data showed that cyclin D1, a pivotal regulator of G1/S phase progression [14], was significantly downregulated by ISO in a dose-dependent manner (Fig. 2A). Furthermore, time course analysis by Western blot revealed that induction of cyclin D1 expression by TPA or EGF was profoundly reduced by 50 μM of ISO at 24 hours in comparison to those in the cells treated with TPA/EGF alone (Figs. 2B and 2C). In contrast, the expression of anti-apoptotic protein XIAP and transcription factor Sp1, which has shown to be suppressed by ISO in human cancer cells [12], was not affected upon ISO treatment in Cl41 cells (Fig. 2A). These data suggested a distinct molecular mechanism of the ISO chemopreventive activity in non-transformed Cl41 cells.

To further prove that ISO downregulation of cyclin D1 protein expression was responsible for the induction of G0/G1 phase arrest and inhibition of cell transformation, Cl41 cells stably transfected with the plasmid encoding FLAG-cyclin D1 T286A mutant, which is refractory to phosphorylation by GSK-3β and thus this point mutation impairs the cyclin D1 proteasomal degradation [15],were established and utilized to test the contribution of cyclin D1 downregulation to the inhibition of cell transformation and induction of G0/G1 phase arrest due to ISO treatment. Overexpression of FLAG-cyclin D1 T286A mutant in the stable transfectant of Cl41 cells was verified as indicated in Fig. 2D. Although ISO treatment did not reduce expression level of FLAG-cyclin D1 T286A mutant, It markedly blocked endogenous cyclin D1 expression (Fig. 2E). As expected, ectopic expression of FLAG-cyclin D1 T286A mutant substantially assisted to bypass ISO-induced G0/G1 phase arrest and attenuated the ISO inhibition of cell transformation induced by TPA or EGF (Fig. 2F-2I), thereby buttressing that ISO-downregulated cyclin D1 expression accounted for its induction of G0/G1 phase arrest and inhibition of cell transformation in Cl41 cells.

### ISO inhibited cyclin D1 transcription via abolishing C-Jun/AP-1 activation

**Figure 3:**
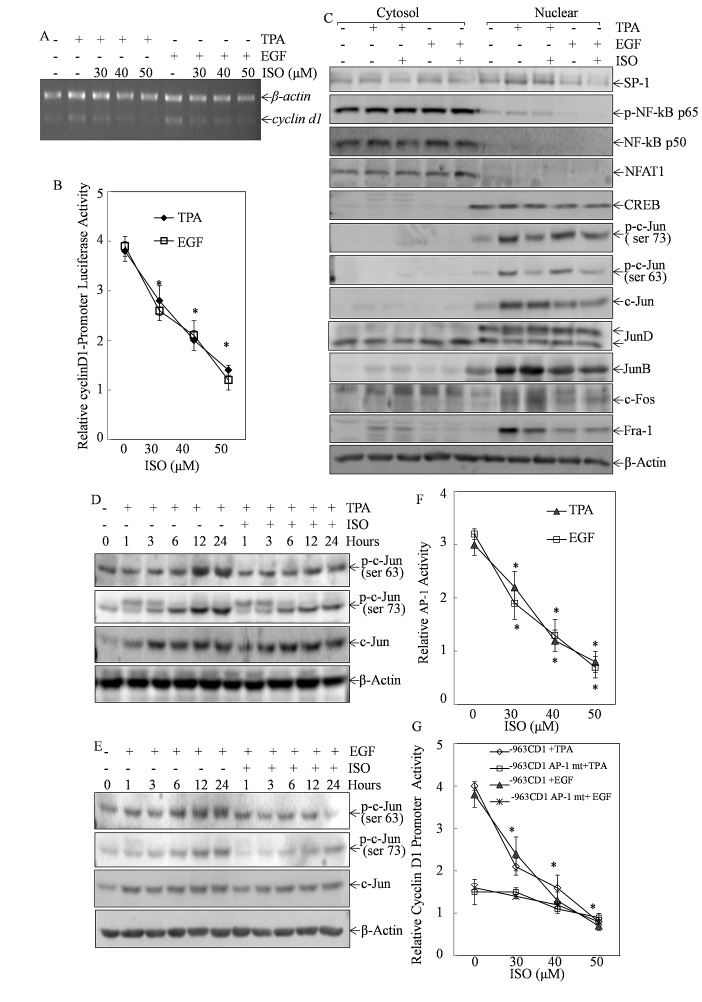
The inhibition of c-Jun/AP-1 by ISO mediated the suppression of cyclin D1 transcription (A) Cl41 cells were pretreated with ISO at the indicated dose for 30 min and then co-incubated with ISO and TPA/EGF (40 ng /ml) for 12 hours. RT-PCR was performed to determine *cyclin d1* mRNA levels. *β-actin* was used as loading control. Data are representative of three independent experiments. (B) Cl41 cells stably transfected with cyclin D1-promoter luciferase reporter were pretreated with ISO in the indicated concentration for 30 min followed by co-incubation with ISO and TPA/EGF (40 ng /ml) for 24 hours. The luciferase activity was measured as described in “Materials and methods”. The results were presented as relative cyclin D1 promoter activity. The symbol (*) indicates a significant decrease as compared with Cl41 cells treated with TPA/EGF alone (*P*<0.05). (C) Cytoplasmic and nuclear extracts from Cl41 cells (treated with TPA/EGF (40 ng /ml) alone, or pretreated with ISO (50 μM) for 30 min and then co-incubated with ISO and TPA/EGF for 9 hours were prepared as described in “Materials and methods”, and then subjected to Western blot analysis with specific antibodies against Sp-1, c-Fos, p-c-Jun (Ser73), p-c-Jun (Ser63), c-Jun, Jun D, Jun B, p-NF-kB p65, NF-kB p50, NFAT1, and CREB, respectively. (D and E) Cl41 cells were treated with TPA/EGF (40 ng/ml), or pretreated with 50 μM ISO for 30 min and then co-incubated with ISO and TPA/EGF for indicated time periods. The p-c-Jun (Ser73), p-c-Jun (Ser63) and c-Jun expression levels were determined by Western blot analysis. (F) AP-1-luciferase reporter containing seven tandem AP-1 binding sites was stably transfected into Cl41 cells. The transfectants were treated with TPA/EGF (40 ng /ml) alone, or pretreated with ISO in the indicated concentrations for 30 min followed by co-incubation with ISO and TPA/EGF for 24 hours. The results were presented as relative AP-1 activity compared to that treated with TPA/EGF alone. The symbol (*) indicates a significant decrease as compared with the indicated cells treated with TPA/EGF alone (*P*<0.05). (G) Cyclin D1 promoter-driven Luciferase reporter (-963CD1) or Cyclin D1 promoter-driven Luciferase reporter with AP-1 binding site mutation (-963CD1 mt) were stably transfected into Cl41 cells. The transfectants were pretreated with ISO in the indicated concentrations for 30 min followed by co-incubation with ISO and TPA/EGF (40 ng /ml) for 24 hours. The luciferase activity was measured as described in “Materials and methods”. The results were presented as relative cyclin D1 promoter activity compared to that treated with TPA/EGF alone. The symbol (*) indicates a significant decrease as compared with the indicated cells treated with TPA/EGF alone (*P*<0.05).

To clarify the underlying mechanism of cyclin D1 downregulation by ISO, we examined *cyclin d1* mRNA level and the transcriptional activity of cyclin D1 promoter after cells were co-treated with ISO and TPA/EGF. RT-PCR analysis and luciferase reporter assay demonstrated that ISO treatment resulted in the reduction of TPA- or EGF-induced both *cyclin d1* mRNA level and its promoter-dependent transcriptional activity in a dose-dependent manner (Figs. 3A and 3B), suggesting that ISO was capable of suppressing cyclin D1 transcription in Cl41 cells.

To identify the transcription factor responsible for ISO downregulation of cyclin D1 transcription, we evaluated the changes in the nuclear translocation of related transcription factors upon ISO treatment for 9 hours. Compared to cells treated with TPA/EGF alone, cells pretreated with 50 μM of ISO displayed the substantial reduction of c-Jun phosphorylation at Ser63 and Ser73 in the nuclear protein extract, whereas c-Jun total protein expression was not affected by ISO (Fig. 3C). Meanwhile, no significant suppression of other transcription factors, including Sp1, NF-kB p65 or p50, NFAT1, CREB, Jun-D, Jun-B, and C-fos was observed following ISO treatment (Fig. 3C). These data suggested that ISO was able to specifically inhibit c-Jun phosphorylation at Ser63 and Ser73. To further substantiate this finding, the inhibitory effect of ISO on c-Jun phosphorylation was examined by means of the time course studies. In comparison to cells treated with TPA/EGF at the corresponding time point, cells with 50 μM ISO pretreatment presented the decreased phosphorylation of c-Jun at Ser63 and Ser73 at approximately 12-24 hours (Figs. 3D and 3E). It was noted that ISO had no impact on c-Jun total expression, although TPA or EGF treatment led to increase in total protein expression (Figs. 3D and 3E).These results together demonstrated that ISO was indeed capable of suppressing c-Jun phosphorylation and activation.

C-Jun is a major component of transcription factor AP-1. We next examined ISO's effect on AP-1 transactivation in Cl41 cells stably transfected with AP-1-luciferase reporter containing seven tandem AP-1 binding sites[16]. As anticipated, ISO treatment significantly blocked AP-1 transactivation in a dose-dependent manner (Fig. 3F). To determine whether ISO-suppressed cyclin D1 transcription was mediated by c-Jun/AP-1, we tested the effect of ISO on cyclin D1 transcription in Cl41 cells stably transfected with luciferase reporter containing cyclin D1 promoters with mutated AP-1 binding sites in compared to that with WT cyclin D1 promoter luciferase reporter. As indicated in Fig. 3G, treatment of cells with either TPA or EGF led to 4 fold induction of cyclin D1 promoter activity in WT cyclin D1 promoter reporter transfectant, and the induction was crippled in the transfectants of AP-1 binding site-mutated cyclin D1 promoter-luciferase reporter. Moreover, co-incubation of cells with ISO exhibited a marked inhibition of cyclin D1 promoter activity in WT cyclin D1 promoter reporter transfectant, whereas it did not show significant effect in AP-1 binding site-mutated cyclin D1 promoter-luciferase reporter (Fig. 3G).Thus, our results strongly implicated that ISO treatment resulted in the suppression of TPA/EGF-induced c-Jun/AP-1 activation, which would in turn inhibit cyclin D1 transcription in Cl41 cells.

### ISO treatment upregulated MKP-1 expression and deactivated MKK7/JNK cascade

**Figure 4:**
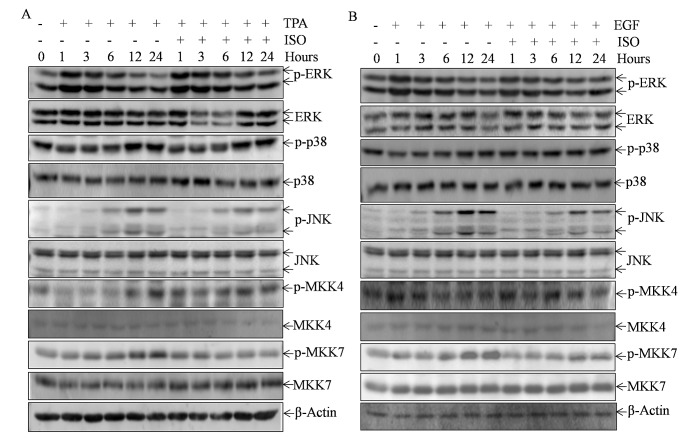
ISO treatment deactivated MKK7/JNK cascade (A and B) Cl41 cells were pretreated with ISO (50 μM) for 30 min and then co-incubated with ISO and TPA/ EGF (40 ng /ml) for indicated time periods. The total cell extracts were subjected to Western blot analysis with specific antibodies against p-ERK, ERK, p-p38, p38, p-JNK, JNK, p-MKK4, MKK4, p-MKK7, and MKK7. β-Actin protein was used as protein loading control. Data are representative of three independent experiments.

The mitogen-activated protein kinases (MAPKs), including ERK, p38, and JNK, have been proven as activators of c-Jun/AP-1 in many cellular settings [17-20]. We thus tested whether TPA/EGF-induced MAPKs activation could be deactivated by ISO in Cl41 cells. The results showed that ISO treatment downregulated the TPA/EGF-induced phosphorylation of JNK at 6-24 hours, but did not affect JNK total expression. By contrast, the induction of phosphorylation of ERK and p38 by TPA/EGF was not impacted by ISO treatment (Figs. 4A and 4B). These data indicated that ISO could selectively deactivate TPA/EGF-induced JNK activation. Given that MKK4 and MKK7 are the two upstream kinases required for JNK full activation [21-23], we next tested whether ISO treatment could likewise attenuate TPA/EGF-induced phosphorylation of MKK4 and MKK7 along the time course. As a consequence, only the obvious reduction of MKK7 phosphorylation was observed when cells were treated with ISO, while MKK7 total expression did not change (Figs. 4A and 4B), thereby suggesting that MKK7 might be a kinase involved in the deactivation of JNK by ISO.

**Figure 5:**
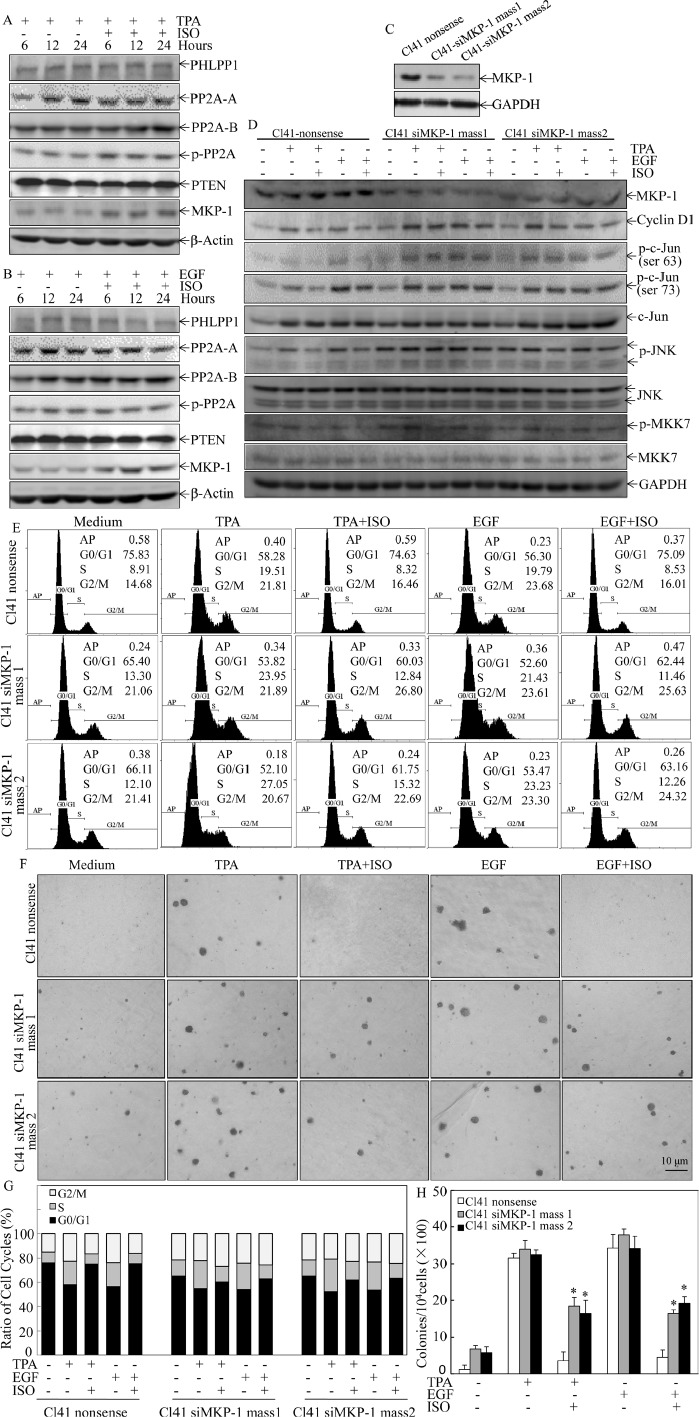
ISO treatment upregulated MKP-1 expression (A and B) Cl41 cells were treated with TPA/EGF (40 ng /ml) alone, or pretreated with ISO (50 μM) for 30 min and then co-incubated with ISO and TPA/EGF for indicated time periods. The cell extracts were subjected to Western blot analysis with specific antibodies against PHLPP1, PP2A-A, PP2A-B, p-PP2A, PTEN, and MKP-1. (C and D) Cl41 cells were transfected with the siMKP-1 or nonsense constructs. Stable transfectants were established and cell extracts were subjected to Western blot analysis with specific antibody against MKP-1(C). The indicated transfectants were treated with medium or TPA/EGF (40 ng /ml) alone, or pretreated with ISO (50 μM) for 30 min and then co-incubated with ISO and TPA/EGF for 24 hours. The cell extracts were subjected to Western blot analysis with specific antibodies against MKP-1, cyclin D1, p-c-JunSer63, p-c-JunSer73, c-Jun, p-JNK, JNK, p-MKK7, and MKK7 (D). (E and G) Cl41 cells were treated with medium or TPA/EGF (40 ng /ml) alone, or pretreated with 50 μM of ISO for 30 min and then co-incubated with ISO and TPA/EGF for 48 hours as described in “Materials and Methods”. Data represent one of three different experiments as indicated in Flow cytometric analysis of cell cycle distribution (E) and percentage of cell-cycle phase (G). (F and H) The cell transformation was determined using the indicated Cl41 stable transfectants in the presence of TPA/EGF (40 ng /ml)with or without ISO (50 μM). Representative images of colonies of transformed cells in soft agar assay were presented (F), and the colonies were counted under microscopy and presented as colonies per 10,000 cells from three independent experiments (H). The symbol (*) indicates a significant increase as compared with that in nonsense transfectants treated with TPA/EGF alone (*P*<0.05).

In addition to kinases, phosphatases might be also involved in JNK deactivation upon ISO treatment. Thus, time course analysis was performed to test whether potential phosphatases are induced by ISO treatment. As shown in Figs. 5A and 5B, ISO treatment upregulated MKP-1 expression, but did not affect the expression of other phosphatases, including PHLPP1, PP2A-A, PP2A-B, and PTEN. Given that MKP-1 is a phosphatase that could directly regulate JNK phosphorylation [24-25], our results indicated that ISO treatment resulted in MKP1 induction, which might subsequently deactivate JNK phosphorylation induced by TPA/EGF.

### MKP-1 knockdown attenuated ISO inhibition of c-Jun/JNK, suppression of cyclin D1 expression, G0/G1 growth arrest and inhibition of cell transformation in Cl41 cells

To further confirm that MKP-1 acted as the precursor regulating the JNK/c-Jun pathway responsible for cyclin D1 downregulation and growth inhibition following ISO treatment, MKP-1 was knocked down in Cl41 cells with a short hairpin RNAi construct that specifically targeted *mkp-1*. Knockdown of MKP1 in two stable mass transfectants was verified in Fig. 5C. As a result, cyclin D1 downregulation and the reduction of phosphorylated c-Jun and JNK by ISO treatment were abrogated in MKP-1 knockdown cells in comparison to that from nonsense transfectant (Fig. 5D). Furthermore, MKP-1 knockdown also reversed ISO-caused G0/G1 cell cycle arrest and inhibition of cell transformation in Cl41 cells (Fig. 5E-5H). Taken together, our results demonstrated a critical role of MKP-1 in the inhibition of the JNK/c-Jun cascade and in turn downmodulates cyclin D1 expression, as well as induction G0/G1 arrest and inhibition of cell transformation following ISO treatment.

### ISO treatment increased *mkp-1* mRNA stability

**Figure 6:**
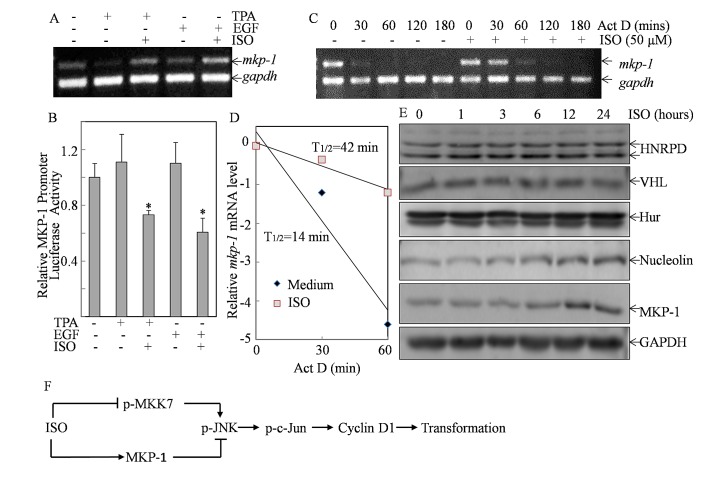
ISO treatment increased *mkp-1* mRNA stability (A) Cl41 cells were treated with medium or TPA/EGF (40 ng /ml) alone, or pretreated with 50μM of ISO for 30 min and then co-incubated with ISO and TPA/EGF for 12 hours. RT-PCR was performed to determine *mkp-1* mRNA levels. The *gapdh* mRNA levels were used as loading control. (B) Cl41 cells stably transfected with MKP-1 promoter luciferase reporter were treated with medium or TPA/EGF (40 ng /ml) alone, or pretreated with 50 μM of ISO for 30 min followed by co-incubation with ISO and TPA/EGF for 24 hours. The luciferase activity was measured as described in “Materials and Methods”. The results were presented as relative MKP-1 promoter activity compared with that of the cells treated with TPA/EGF. The symbol (*) indicates a significant decrease as compared with cells treated with TPA/EGF alone (*P*<0.05). (C) Cl41 cells were pretreated with ISO (50 μM) for 6 hours and then co-incubated with ISO and actinomycin D (20 μM) for indicated time periods. Total RNA was isolated and RT-PCR was then performed to determine mkp-1 mRNA levels. The result was a representative one from three independent experiments. (D) The relative mRNA level of *mkp-1* was determined by ImageQuant 5.2 (GE Healthcare). Natural logarithm of ratio [*mkp-1*]_t_/[*mkp-1*]_0_ was plotted against the time and the half life of *mkp-1* mRNA was calculated via linear regression. (E) Cl41 cells were treated with ISO (50 μM) for indicated time periods. The total cell extracts were subjected to Western blot analysis with specific antibodies against HNRPD, VHL, Hur, Nucleolin, and MKP-1. (F) The diagram indicates mechanisms responsible for ISO inhibition of cell transformation.

To elucidate mechanism underlying ISO upregulation of MKP-1 expression, we determined *mkp-1* mRNA level following co-incubation of ISO with either TPA or EGF. The results revealed that *mkp-1* mRNA level was inhibited in Cl41 cells following either TAP or EGF treatment (Fig. 6A). However, this inhibition by TPA or EGF was completely reversed to the level that is even higher than basal level by co-incubation of cells with ISO, suggesting that ISO upregulated MKP-1 expression at either transcription or mRNA stability. To address this question, the MKP-1 promoter-driven luciferase reporter was transfected into Cl41 cells and the MKP-1 promoter activity in the transfectant was determined in the cells treated with ISO followed TPA or EGF. As shown in Fig. 6B, ISO treatment significantly downregulated MKP-1 promoter activity, suggesting ISO treatment might upregulate MKP-1 expression via increase *mkp-1* mRNA stability. This notion was greatly supported by the results showing that pretreatment of cells with ISO for 6 hours followed by the treatment of actinomycin D for 20 min increased *mkp-1* mRNA half-life (T_1/2_ increased to 42 min) in comparison to that observed in cells treated with actinomycin D alone (T_1/2_=14 min) (Figs. 6C& 6D). Thus, our data proved that ISO treatment stabilized *mkp-1* mRNA stability.

Several RNA-binding proteins, e.g. HNRPD, VHL, Hur, and Nucleolin, have been reported to bind their target mRNA and increased mRNA stability [26-28]. We hence tested whether any of these proteins was involved in ISO upregulation of *mkp-1* mRNA stability. Western blot analysis showed that ISO treatment could clearly increase the Nucleolin expression, and moreover Nucleolin protein upregulation was associated with the MKP-1 protein induction along the time course studies (Fig. 6E), suggesting that Nucleolin might participated in the increased *mkp-1* mRNA stability following ISO treatment.

## DISCUSSION AND CONCLUSIONS

The naturally occurring compound ISO [8] has been proved to possess anti-cancer activities against multiple human cancer cell lines [12]. Mechanistic studies revealed that ISO downregulation of XIAP and cyclin D1 protein expression accounted for its anti-cancer effects in human cancer cells [12-13]. In the current study, we investigated potential cancer chemopreventive activity of this phytochemical by exploring its potential inhibitory effect on cell transformation in Cl41 cells. Our results demonstrated that ISO treatment was capable of inhibiting TPA/EGF-induced colony formation via upregulating MKP-1 expression and arresting G1/S cell-cycle progression, whereas it did not produce redundant cytotoxic effects on normal non-transformed cells, thereby suggesting the cancer chemopreventive activity of ISO. Further study indicated that ISO-upregulated MKP-1 expression mediated the inhibition of JNK/C-Jun/AP-1 activation and in turn led to cyclin D1 downregulation, G1/S cell-cycle arrest, and cell transformation inhibition. Therefore, we identified a novel function of ISO as a cancer chemopreventive agent that is mediated through a molecular mechanism distinct from that responsible for ISO cancer therapeutic effect.

Abnormal expression of cyclin D1 has been reported in many human cancers [29]. Particularly, *cyclin d1* mRNA amplification has been observed in approximately 45% breast carcinomas [30]. It is also reported that more than 50% patients presented cyclin D1 overexpression in a total of 307 patients with non-melanocytic skin cancer [31]. Cyclin D1 has been shown to play a crucial role in cancer development, especially skin carcinogenesis [32-33]. Transgenic mice with conditional cyclin D1 overexpression are much easier to develop skin tumor when exposed to dimethylbenz[a]anthracene [33]. Also, cyclin D1 knockdown was capable of attenuating arsenite-induced cell transformation in mouse Cl41 cells and human HaCat cells [34-35]. Hence, cyclin D1 has been regarded as an important player in cancer development as well as a target for cancer prevention and therapy [36]. Our previous study, for example, has reported that suberoylanilide hydroxamic acid (SAHA) is able to inhibit EGF-induced cell transformation through reduction of *cyclin d1* mRNA stability [37]. In the present study, we found that ISO inhibition of cell transformation and cell cycle progression in Cl41 cells was mediated through downregulation of cyclin D1 protein expression. Further investigation proved that ISO regulated cyclin D1 through inhibiting its transcription, and this process was mediated through the suppression of its transcription factor c-Jun/AP-1 activation via a novel mechanism, i.e. upregulating MKP-1 expression.

MKK7/JNK/AP-1 cascade is an important oncogenic signaling pathway [1, 38]. Murine genetic loss-of-function studies have demonstrated that targets of JNK signaling cascade, particularly c-Jun and c-fos, may contribute to chemical-induced murine epidermal neoplasia [39-41]. JNK/AP-1 has been reported to play a role in inducing aberrant cell proliferation associated with human skin carcinogenesis [38-43]. Moreover, previous studies have also reported that TNFR1/MKK7/JNK/AP-1 cascade promoted human neoplasia and inhibition of this pathway represented a potential therapeutic approach for squamous cell carcinoma (SCC) [38]. Therefore, the discovery in the current investigation that ISO could inhibit the activation of MKK7/JNK/c-Jun/AP-1 cascade triggered by TPA/EGF in Cl41 mouse epidermal cells reveals the application of this phytochemical for the cancer prevention.

In addition to MKK7, phosphatase MKP-1 was also shown to be upregulated and mediate JNK deactivation following ISO treatment. Further results indicated that ISO treatment could augment *mkp-1* mRNA stability. Yet, although our data suggested that Nucleolin might be involved in the stabilization of *mkp-1* mRNA, which has never been reported before, the detailed mechanism underlying this process remains elusive and thus is currently underway of investigation in our laboratory. On the other hand, it is intriguing that albeit with profoundly decreased promoter activity, *mkp-1* mRNA level was upregulated following ISO treatment. This seemingly paradox might be due to fact that MKP-1 is regulated by c-Jun, which has been reported in sympathetic neurons [44] and thus c-Jun deactivation might result in the downregulation of MKP-1 promoter transcriptional activity following ISO treatment. But the detailed mechanism responsible for ISO downregulation of MKP-1 transcription in Cl41 cells is still under investigation.

In summary, our studies demonstrated that ISO exerted an inhibitory effect of TPA/EGF-induced cell transformation with induction G0/G1 cell-cycle arrest in Cl41 cells. Mechanistic insight into cell-cycle arrest induction suggested that this inhibitory effect is through MKP-1 upregulation and MKK7 deactivation, both of which would concertedly deactivate JNK/c-Jun cascade, thereby leading to cyclin D1 downregulation (Fig. 6F). These findings implicate the potential utilization of ISO as a cancer chemopreventive agent.

## MATERIAL AND METHODS

### Cell Culture and Reagents

Mouse epidermal Cl41 cells and their transfectants were cultured in 5% Fetal bovine Serum (FBS) MEM containing with 1% penicillin/streptomycin and 2 mM L-glutamine (Life Technologies). Cells were maintained in a humidified incubator at 37°C with 5% CO_2_ atmosphere[45]. The antibodies specific against phospho-c-Jun, c-Jun, phospho-JNKs, JNKs, phospho-ERKs, ERKs, phospho-p38, p38, phospho-MKK7, MKK7, phospho-MKK4, MKK4, PP2A A subunit, XIAP, SP1, β-Actin and GAPDH were purchased from Cell Signaling Technology Inc (Beverly, MA). The antibodies specific against cyclin D1, c-Fos, CREB, Fra1, p-NF-kB p65, NF-kB p50, JunD, JunB, PTEN and MKP-1 were bought from Santa Cruz Biotechnology (Santa Cruz, CA). The antibody against FLAG was obtained from Covance Inc. (Princeton, NJ). The antibodies against phospho-PP2A and PP2A B subunit were purchased from Epitomics (Burlingame, CA). Actinomycin D was purchased from Calbiochem (Billerica, MA). ISO with over 98% purity was purchased by Higher Biotech (Shanghai, China). The preparation of ISO was performed as described in our previous studies [12].

### Plasmids and Cell Transfection

The -963cyclin D1 promoter-driven luciferase reporter and its AP-1 binding site mutant were kindly provided by Dr. Richard G Pestell (Thomas Jefferson University Jefferson Medical College) [46-47]. The cyclin D1 promoter region containing -963 to +14 was subcloned into the pA3LUC and named as the -963 cyclin D1 promoter-driven Luciferase reporter (-963CD1). The -963 CD1AP-1 mt was derived from -963CD1 with two nucleotide mutation within AP-1 binding site of the cyclin D1 promoter. The MKP-1 promoter-driven luciferase reporter was a gift from Dr. Yusen Liu (National Institutes of Health) [48]. Cyclin D1 promoter-driven luciferase reporter was described in our previously study [49]. The AP-1-driven luciferase reporter containing seven tandem AP-1 binding sites (TGACTAA) was purchased from Stratagene (La Jolla, CA). FLAG–cyclin D1 T286A/pCMV5 expression vector was a gift of Dr. Udit N. Verma (University of Texas Southwestern Medical Center at Dallas) [50]. The specific small-interference RNA (siRNA) vector targeted mouse *mkp1 (dusp1)* was purchased from Sigma-Aldrich Chemical (St. Louis, MO). Stable co-transfections were performed with specific cDNA constructs together with pSUPER-puro vector using PolyJet™ DNA In Vitro Transfection Reagent (SignaGen Laboratories, Gaithersburg, MD) according to the manufacturer's instructions. For stable transfection selection, cultures were subjected to puromycin selection for 4–6 weeks, and surviving cells were pooled as stable mass transfectants. These stable transfectants were cultured in the selected antibiotic-free medium for at least two passages before utilization for experiments[51-53].

### Anchorage-independent Growth Assay

Soft agar assay was performed as described previously [54]. Briefly, 2.5 ml of 0.5% agar in basal modified Eagle's medium (BMEM) supplemented with 10% FBS with or without ISO, EGF, TPA, EGF+ISO or TPA+ISO respectively was layered onto each well of 6-well tissue culture plates. 1×10^4^ Cl41 cells or their transfectants were mixed with 1 ml of 0.5% agar BMEM supplemented with 10% FBS with or without EGF/TPA and ISO, and then layered on top of the 0.5% agar layer. The plates were incubated at 37 °C in 5% CO2 for 3 weeks. The colonies with more than 32 cells were scored and the results were presented as colonies/10^4^ cells[45, 47, 51].

### Flow Cytometry Assay

Cells (1×10^5^) were cultured in each well of 6-well plates till 60-70% confluence with normal culture medium. Cells were synchronized by the replacing the medium containing 0.1% FBS for 24 hours, and then replacing medium containing 1% FBS with or without EGF/TPA and ISO, and cultured for another 24 hours as indicated in figure legends. The cells were harvested and fixed with 3 ml of ice-cold 80% ethanol overnight. The fixed cells were washed twice with PBS, and then suspended in Propidium Iodide staining solution (Propidium Iodide 50 mg /ml, RNAse A 10 mg /ml, and 0.1% Triton X-100) (Sigma-Aldrich Chemical, St. Louis, MO) for at least 1 hour at 4°C. The DNA content was determined by means of Flow Cytometric Analysis with the Epics XL FACS (Beckman Coulter, Miami, FL) and EXPO 32 software as described previously [45, 51, 55].

### Cell Proliferation Assay

Confluent monolayers of Cl41 cells were trypsinized and 2×10^3^ viable cells suspended in 100 μl MEM supplemented with 5% FBS were added to each well of 96-well plates. After adherent, cells were synchronized by replacing the medium containing 0.1% FBS for 24 hours, and then replacing culture medium containing 1% FBS with or without ISO, and cells treated with ISO at indicated doses were cultured for 48 hours. The viability of the cells was determined using CellTiter-Glo® Luminescent Cell Viability Assay kit (Promega, Madison, WI) with a luminometer (Wallac 1420 Victor 2 multipliable counter system). The results were presented as proliferation index (relative luminescence signal to medium control)[56-57].

### Western Blotting

Cell extracts were prepared with cell lysis buffer (10 mM Tris-HCl, pH 7.4, 1% SDS, and 1 mM Na_3_VO_4_) and protein concentrations were determined by NanoDrop 1000 spectrophotometer (Thermo Scientific, Wilmington, DE). 30~80 μg of protein sample from the cell extracts were separated on SDS-polyacrylamide gels (SDS-PAGE), transferred, and probed with indicated antibody. The protein band that was specifically bound to the primary antibody was detected using an alkaline phosphatase-linked secondary antibody and an ECF Western blotting system (Amersham Biosciences, Piscataway, NJ)[47, 58-59].

### RT-PCR

Total RNA was extracted with TRIzol reagent (Invitrogen, Carlsbad, CA), and cDNAs were synthesized with the ThermoScript RT-PCR system (Invitrogen, Carlsbad, CA). The mouse *cyclin d1* cDNA fragments were amplified by primers 5'-TCCCTTGACTGCCGAGAAG-3' and 5'-AGACCAGCCTCTTCCTCCAC-3'. The primers for mouse *mkp-1* are 5'-GAA GCG TTT TCG GCT TCC TG-3' and 5'- AGG TAA GCA AGG CAG ATG GTG-3'. The primers for mouse *gapdh* are 5'-GGA GGT TGT CAT CCC TCAGA-3' and 5'-TCC TCC TCA GCC ACA CTC TT-3'. The primers for mouse *β-actin* are 5'-ATA TCG CTG CGC TGG TCG TC-3' and 5'-AGG ATG GCG TGA GGG AGA GC-3'. The PCR products were separated onto 3% agarose gels, stained with EB, and scanned the images from a UV light as described previously[45, 52, 60-61].

### Luciferase Assay

Cl41 cells were transfected with the indicated luciferase reporter construct in combination with the pRL-TK vector (Promega, Madison, WI) as an internal control. The transfectants were seeded into 96-well plates. After the cell density reached 70-80%, cells were treated as indicated in the figure legends, and were then extracted with luciferase assay lysis buffer (Promega, Madison, WI). The luciferase activity was determined by the microplate luminometer LB 96V (Berthold GmbH & Co. KG, Bad Wildbad, Germany) using the luciferase assay system (Promega Corp., Madison, WI) as described [53, 61-62].

### Nuclear Extract Preparation

Preparation of nuclear extracts was assessed as previously described [62]. Cl41 cells were plated into 10-cm culture dishes at 80% confluence, treated with EGF/TPA alone or pre-treated with 50 μM ISO for 30 mins and then co-treated with EGF or TPA for 9 hours. The nuclear proteins were extracted according to the protocol of the Nuclear/Cytosol Fractionation Kit (BioVison Technologies, Mountain View, CA). Equal protein concentrations were determined using a protein quantification assay kit (Bio-Rad, Hercules, CA). Nuclear extracts were stored at −80 °C until they were used.

### Statistical Analysis

The student's t-test was used to determine the significance between treated and untreated group. The results are expressed as mean ± SD from at least three independent experiments. *P*<0.05 was considered as a significant difference between compared groups.
